# The bHLH Transcription Factor, Hairy, Refines the Terminal Cell Fate in the *Drosophila* Embryonic Trachea

**DOI:** 10.1371/journal.pone.0014134

**Published:** 2010-11-30

**Authors:** Yaoyao Zhan, Saw W. Maung, Bing Shao, Monn Monn Myat

**Affiliations:** Department of Cell and Developmental Biology, Weill Medical College of Cornell University, New York, New York, United States of America; Stockholm University, Sweden

## Abstract

**Background:**

The pair-rule gene, *hairy*, encodes a basic helix-loop-helix transcription factor and is required for patterning of the early *Drosophila* embryo and for morphogenesis of the embryonic salivary gland. Although *hairy* was shown to be expressed in the tracheal primordia and in surrounding mesoderm, whether *hairy* plays a role in tracheal development is not known.

**Principal Findings:**

Here, we report that *hairy* is required for refining the terminal cell fate in the embryonic trachea and that *hairy*'s tracheal function is distinct from its earlier role in embryonic patterning. In *hairy* mutant embryos where the repressive activity of *hairy* is lost due to lack of its co-repressor binding site, extra terminal cells are specified in the dorsal branches. We show that *hairy* functions in the muscle to refine the terminal cell fate to a single cell at the tip of the dorsal branch by limiting the expression domain of *branchless* (*bnl*), encoding the FGF ligand, in surrounding muscle cells. Abnormal activation of the Bnl signaling pathway in *hairy* mutant tracheal cells is exemplified by increased number of dorsal branch cells expressing Bnl receptor, Breathless (Btl) and Pointed, a downstream target of the Bnl/Btl signaling pathway. We also show that *hairy* genetically interacts with *bnl* in TC fate restriction and that overexpression of *bnl* in a subset of the muscle surrounding tracheal cells phenocopied the *hairy* mutant phenotype.

**Conclusions/Significance:**

Our studies demonstrate a novel role for Hairy in restriction of the terminal cell fate by limiting the domain of *bnl* expression in surrounding muscle cells such that only a single dorsal branch cell becomes specified as a terminal cell. These studies provide the first evidence for Hairy in regulation of the FGF signaling pathway during branching morphogenesis.

## Introduction

Epithelial morphogenesis is a prevalent process necessary for the formation of many essential organs during embryogenesis. While some tubular organs, such as the vasculature, lung and kidney are branched structures, others, such as the gut and neural tubes are unbranched. Through the use of genetically amenable model organisms, such as *Drosophila* and Zebrafish, we are beginning to unravel the mechanisms of epithelial branching morphogenesis; however, it is still not clear why some tubular organs branch whereas others do not. A key signaling pathway that controls branching morphogenesis in both vertebrate and invertebrate organs is the fibroblast growth factor (FGF) pathway [1]. For example, loss of FGF signaling in the mammalian lung or the *Drosophila* trachea severely disrupts branching morphogenesis in these organs [2]–[7].

Studies in the *Drosophila* embryonic trachea have contributed significantly to our understanding of branching morphogenesis. The embryonic trachea is an interconnected network of branched epithelial tubes that becomes functional during the larval stage to transport oxygen and other gases throughout the organism. The pattern of the larval trachea is established during embryogenesis when cells from ten tracheal placodes on each side of the embryo invaginate into the underlying mesoderm and then migrate out in a distinct pattern to form the primary branches. During the initial outgrowth of the tracheal primary branches, tracheal cells expressing the FGF receptor, Breathless (Btl), migrate in response to the FGF ligand, Branchless (Bnl), which is expressed in discrete clusters of non-tracheal cells that surround the migrating tracheal cells [5], [6], [8], [9]. Later in embryogenesis, *bnl* expression confers secondary cell fates, such as the terminal cell fate, to cells at the tip of the growing branches [5], [6], [10], [11]. Thus, Bnl/Btl signaling is required throughout tracheal development for initial migration and outgrowth of the primary branches as well as for specification of the secondary cell fates. One mechanism by which Bnl/Btl signaling is sustained in tracheal cells is through a positive feedback loop, whereby Bnl/Btl signaling activates MAP-kinase and the ETS-domain transcription factor, Pointed, to induce late *btl* expression [12].

During migration of primary tracheal branches, markers, such as *pointed* and *sprouty*, that define the tips of migrating branches are expressed broadly, only to become restricted to a single cell later [10], [13]. This suggested that all tracheal cells are initially equivalent but then specific cell fates become restricted through regulation of gene expression. In the dorsal branch, which typically consists of five or six cells, one cell at the branch tip adopts the terminal cell fate and branches extensively to deliver oxygen to neighboring tissues whereas a second cell at the branch tip adopts a fusion cell fate and mediates fusion of tracheal branches from adjacent hemisegments and contralateral branches at the dorsal midline [13], [14]. Genetic mosaic analysis showed that tracheal cells at the tips of migrating branches compete with each other such that cells with the highest Bnl/Btl signaling activity become the “lead” cell which is then specified to be the terminal cell, whereas those with less signaling activity become the “follower” stalk cells of the tube [4]. FGF signaling induces Notch (N) signaling through activation of the N ligand, Delta (Dl). Activated Dl in the tip cells then activates N in neighboring stalk cells to restrict the fusion and terminal cell fates [15]–[17]. Thus, FGF signaling not only regulates tracheal cell migration, but also restricts cell fates via N-mediated lateral inhibition.

*hairy* is a pair-rule gene whose role in early patterning of the *Drosophila* embryo is well established [18], [19]. Hairy belongs to a small family of bHLH transcription factors related to the HES/HESR/HRT/HEY proteins in mammals [20]–[22] and Gridlock in Zebrafish [23]. Hairy and its related proteins generally function as transcriptional repressors which are expressed in various tissues and regulate key developmental events such as cardiovascular development [21], [24], [25]. We previously showed that loss of *hairy* function results in expansion and branching of the normally unbranched embryonic salivary gland without excess cell proliferation [26]. We further showed that *hairy* controls salivary gland lumen size and shape by regulating the extent of apical membrane generation through negative regulation of the transcription factor, Huckebein (Hkb) and its downstream target genes, *crumbs*, that encodes an apical membrane determinant, and *klarsicht* that encodes a putative minus-end directed microtubule motor [26]. Hairy was previously reported to be expressed in the invaginating embryonic trachea as well as in the surrounding visceral and somatic mesoderm [18], [19]; however, the role Hairy plays in tracheal development is not known. Here, we demonstrate a novel function for *hairy* in refining the terminal cell fate at the tips of primary tracheal branches.

## Results

**Figure 1:**
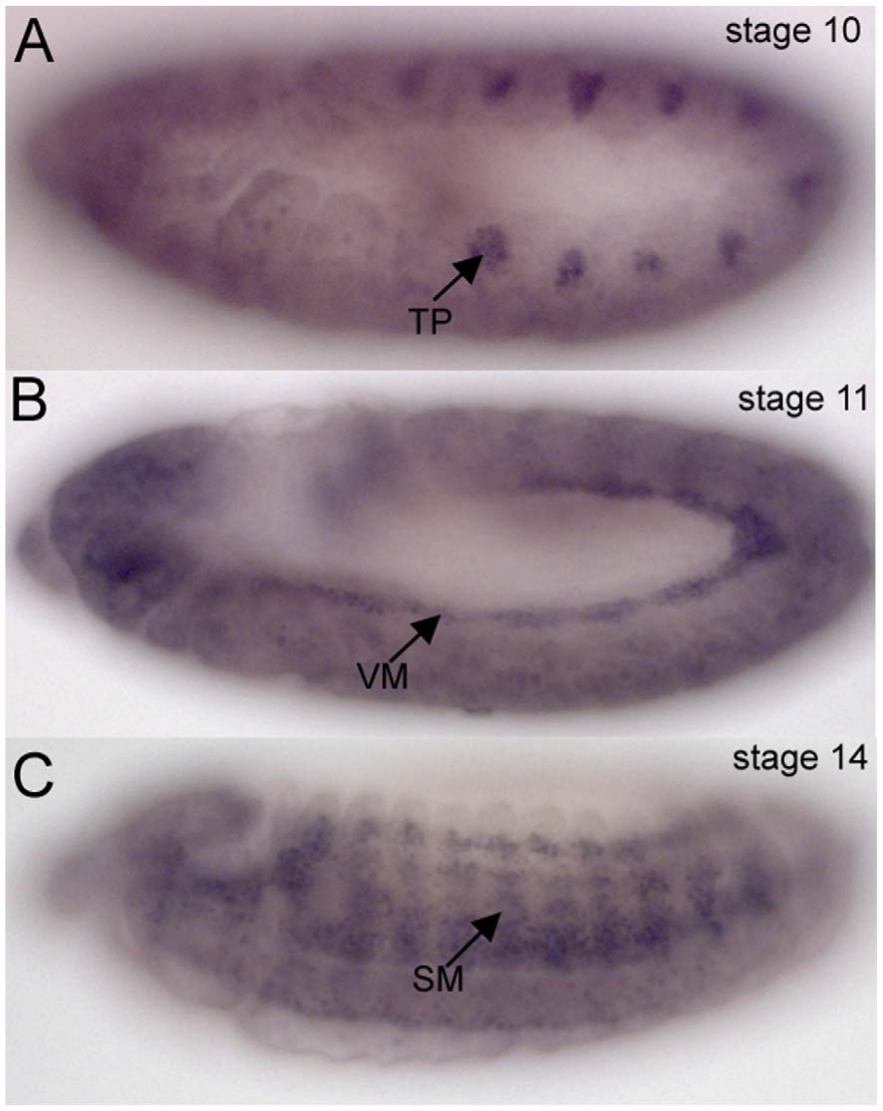
Hairy protein expression during embryogenesis. In wild-type embryos, Hairy protein is expressed in invaginating cells of the tracheal placode (TP) (A, arrow), in the visceral mesoderm (VM) (B, arrow) and somatic mesoderm (SM) (C, arrow). Scale bar A represents 20 µm.

Hairy protein was first detected in the tracheal placodes prior to and during invagination but not in the internalized tracheal cells (Figure 1A). In addition to tracheal expression, Hairy protein was also expressed in the visceral and somatic mesoderm, as previously reported (Figure 1B and C) [18], [19]. To understand *hairy* function in tracheal development, we analyzed an allelic series of *hairy* mutants, including *h^22^*, *h^674^, h^47^, h^m2^, h^1J3^* and *h^1^*, for tracheal defects. *h^22^* is a lethal EMS-induced null allele encoding a truncated protein of only 42 amino acids due to a premature stop codon; *h^674^* is a lethal EMS-induced hypomorph allele encoding a protein lacking the C-terminal 103 amino acids due to a premature stop codon and *h^1J3^* and *h^1^* are viable alleles with inserted transposable elements ([26], [27]; Flybase). Although the exact molecular lesion of *h^47^* and *h^m2^* are not known, they are reported to have no segmentation defects and to fully complement the segmentation and lethality of strong *hairy* alleles [28]. Consistent with their molecular lesions, the *hairy* pair-rule phenotype was most severe in *h^22^* embryos compared to *h^1J3^* and *h^1^*embryos which showed no patterning defect (data not shown). *h^674^* mutant embryos showed a range of segmentation defects, though none as severe as that of *h^22^* mutant embryos [26].

**Figure 2:**
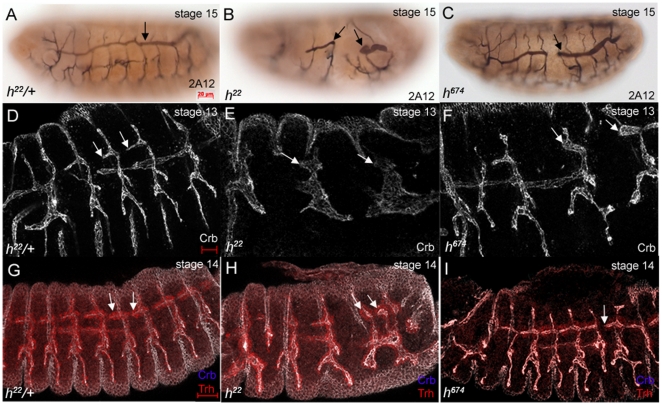
Tracheal development is defective in *hairy* mutant embryos. In *h^22^* heterozygous embryos, the dorsal trunk (DT) is a continuous tube (A, arrow) whereas in *h^22^* (B) and *h^674^* (C) homozygous embryos, the DT is broken (B and C, arrows). In *h^22^* heterozygous embryos (D and G), tracheal cells migrate to form six primary branches (D and G, arrows). In *h^22^* (E and H) and *h^674^* (F and I) homozygous embryos, tracheal cells fail to migrate out (E, F, H and I, arrows). Embryos in panels A, B and C were double stained for 2A12 and β-galactosidase (β-gal) to distinguish heterozygous from homozygous siblings. Embryos in panels D to F were stained for Crumbs (Crb) to label the lumen and β-gal (not shown), and embryos in panels G to I were stained for Crb (white), β-gal (not shown) and Trachealess (Trh) (red) to label tracheal nuclei. Panels in D to I are projections of confocal sections of laterally viewed embryos. Scale bars in D and G represent 10 µm.

**Figure 3:**
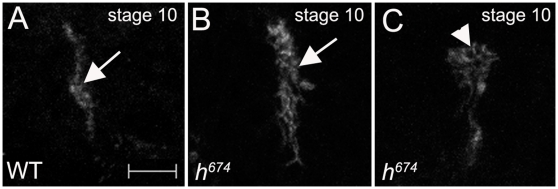
Hairy is required for proper invagination. In wild-type embryos (A), invaginating tracheal cells form a finger-like structure. In some *h^674^* mutant embryos (B), invaginating tracheal cells form a finger-like structure, whereas in other mutant embryos (C), one end of the structure was widened (C, arrowhead). Embryos in A–C were stained for Crb and β-gal (not shown). All panels of embryos are lateral views. Scale bar in A represents 5 µm.

In embryos homozygous for either *h^22^*or *h^674^*, all tracheal cells invaginated (Figure 2H and I), indicating that although *hairy* is expressed in invaginating tracheal cells loss of *hairy* function did not prevent tracheal cells from being internalized. However, *hairy* may still play a role in tracheal invagination since recent studies showed that tracheal cells can still be internalized in mutants where certain features of invagination are defective [29], [30]. In some *h^674^* mutant embryos, invaginating tracheal cells formed the previously reported “finger-like structure” [29] as in wild-type embryos, whereas in other mutant embryos, one or both ends of the structure was widened indicative of uncoordinated invagination (Figure 3A–C). Thus, during early tracheal development, *hairy* is required for coordinated invagination.

Although all tracheal cells were internalized in *h^22^*and *h^674^*mutant embryos, later tracheal development was defective. In stage 15 *h^22^* and *h^674^*mutant embryos, the dorsal trunk (DT) was broken and did not form a continuous tube (Figure 2B and C). Discontinuity of the DT was observed in 96% of *h^22^* mutant DTs (n = 125) and 53% of *h^674^*mutant DTs (n = 237) analyzed. *h^1^* and *h^IJ3^* mutant embryos did not show defects in formation of the primary tracheal branches (data not shown); however, in embryos *trans*-heterozygous for *h^22^* and *h^1J3^*, the DT was broken (data not shown). *h^m2^* and *h^47^* mutant embryos, which had no segmentation defects, formed a normal tracheal network (data not shown). During earlier stages of tracheal migration, *h^22^* mutant cells failed to migrate out and instead formed large clusters of internalized cells (Figure 2E and H). Tracheal migration defects were milder in *h^674^*mutant embryos although delayed migration of DT cells was apparent (Figure 2F and I). We confirmed that DT migration defects in *hairy* mutant embryos were not due to a failure to specify DT identity by staining for the DT marker, *spalt*; in both *h^22^* and *h^674^* mutant embryos, *spalt* was expressed in the same temporal and spatial manner as heterozygous siblings (data not shown).

### Hairy function in terminal cell specification

**Figure 4:**
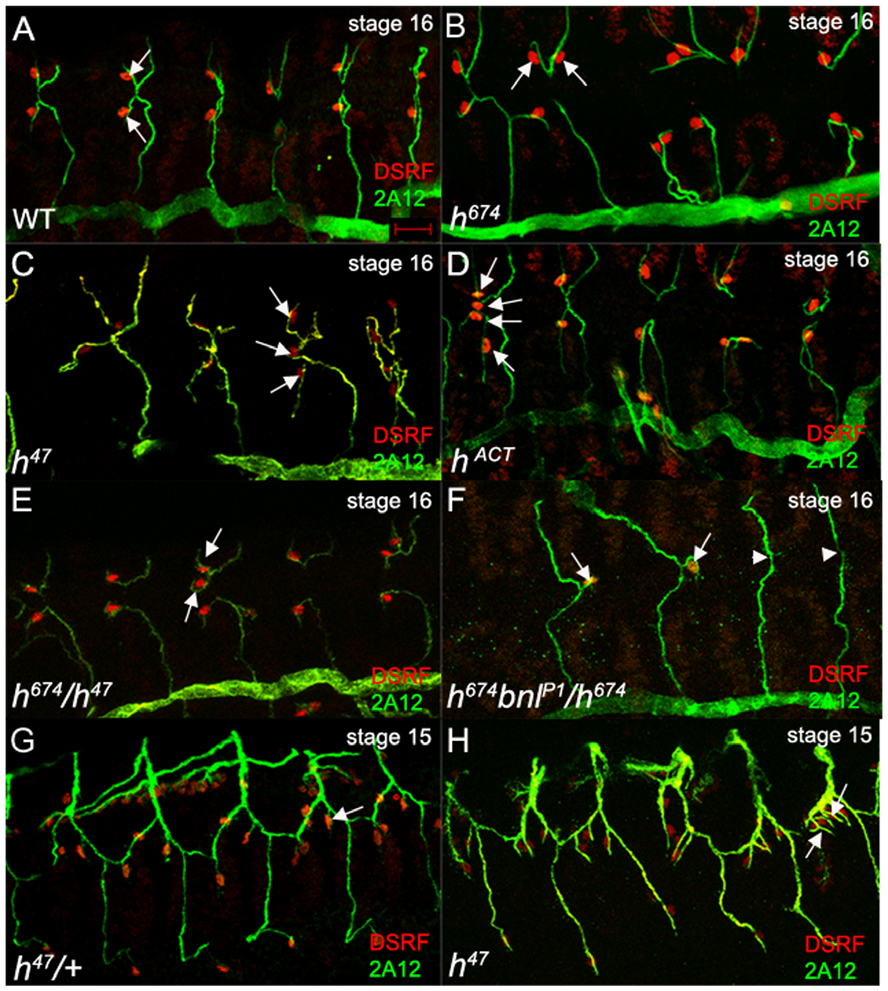
Hairy limits terminal cell number. In WT embryos (A), a single terminal cell (TC) is specified at the tip of each dorsal branch (A, arrows). In *h^674^* (B), *h^47^* (C) and *h^ACT^* (D) mutant embryos and embryos *trans*-heterozygous for *h^674^* and *h^47^* (E), two or more TCs are specified per dorsal branch (B, C, D and E, arrows). In *h^674^* homozygous embryos that are also heterozygous for *bnl^P1^*(F), some DBs have a TC (F, arrows) whereas other DBs are missing TCs (F, arrowheads). Extra terminal cells are specified in lateral trunks of *h^47^* homozygous embryos compared to heterozygous siblings (G and H, arrows). Embryos in A–F are at stage 16 and embryos in G and H are at stage 15. All embryos were stained for DSRF (red) to label TCs and 2A12 (green) to label the tracheal lumen. Panels A–E show projections of confocal sections of dorsal view embryos whereas those of G and H are projections of lateral view embryos. Scale bar in A represents 20 µm.

Due to *hairy*'s well-established role in embryonic patterning, we limited our analysis of *hairy* function in tracheal development to *h^47^* mutant embryos which did not exhibit any segmentation defects [28]and *h^674^* embryos which have mild or no segmentation defects. In wild-type embryos, a single cell at the distal tip of each dorsal branch becomes specified as the terminal cell (TC) (Figure 4A). In contrast, in *h^47^* and *h^674^* homozygous embryos and *trans*-heterozygous embryos of *h^47^* and *h^674^*, extra TCs were found at the tips of the DBs (Figure 4B, C and E); extra TCs were also detected in the lateral trunk branches of *h^47^* homozygous embryos compared to heterozygous siblings (Figure 4G and H). To eliminate the possibility that specification of extra TCs observed in *h^674^* mutant trachea could be a secondary consequence of *hairy*'s patterning role in the early embryo, we analyzed embryos expressing a heat shock-induced activated form of Hairy (Hairy^ACT^) where the transcriptional activation domain of the herpes simplex virus VP16 protein is fused to a truncated Hairy protein that retains the bHLH and helical/Orange domains but lacks the C-terminal WRPW motif required for repression by *hairy* [31]. Hairy^ACT^ (h^ACT^) has been shown to act as an activator instead of a repressor by promoting transcription of specific target genes [31]. Heat shock-induced expression of h^ACT^ at eight hours of embryogenesis after segmentation of the early embryo is complete resulted in a properly segmented embryo with an intact tracheal network (data not shown); however, in terms of TC specification, h^ACT^-expressing embryos phenocopied the *hairy* mutant phenotype with extra TCs being specified at the tips of the DBs (Figure 4D). The extra TCs specified in h^ACT^ expressing embryos that were properly segmented confirmed that *hairy* is normally required for refining the TC fate to a single cell and that this tracheal function of *hairy* is independent of its earlier patterning role.

**Figure 5:**
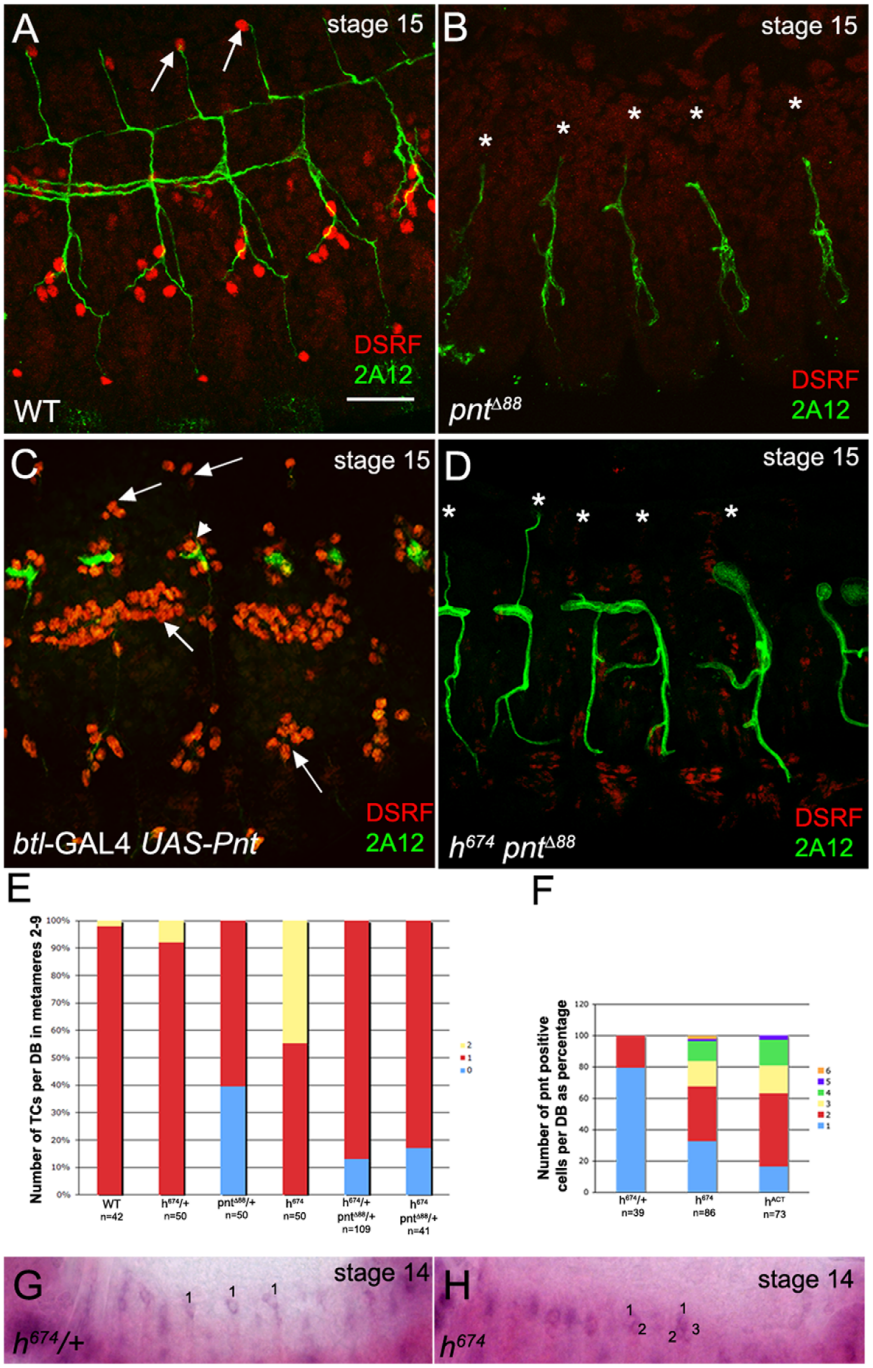
Pointed is epistatic to Hairy in terminal cell specification. In WT embryos (A), a single TC is specified in each dorsal branch (A, arrows), whereas dorsal branches of *pnt^Δ88^* homozygous embryos lack TCs (*, B). In embryos overexpressing wild-type *pnt* in the entire trachea with *btl*-GAL4 (C), extra TCs are specified in all tracheal branches (C, arrows), including the dorsal trunk (C, arrowhead). Dorsal branches of embryos homozygous for both *h^674^* and *pn^Δ88^* lack TCs (*, D). (E) Graph shows number of TCs per dorsal branch in metameres 2–9 in WT, *h^674^* and *pnt^Δ88^* mutant embryos; n represents number of embryos scored. (F) Graph shows percentage of dorsal branches with various numbers of *pnt* RNA expressing cells; n represents number of embryos scored. *h^674^* heterozygous embryos (G) have a single dorsal branch cell expressing *pnt* RNA (G), whereas homozygous siblings have extra dorsal branch cells expressing *pnt* RNA (H). Embryos shown in panels A–D were stained for DSRF (red), 2A12 (green) and β-gal (not shown). Embryos shown in G and H were hybridized *in situ* for *pnt* RNA (purple) and *lac-Z* (not shown). Scale bar in A represents 20 µm.

Formation of terminal branches requires the ETS domain transcription factor, Pointed (Pnt) [13]. In embryos homozygous for a null allele of *pnt*, *pnt^Δ88^*, TCs were not specified unlike in wild-type embryos (Figure 5A and B). In contrast, overexpressing wild-type *pnt* in the entire trachea with *btl*-GAL4 led to specification of extra TCs in all tracheal branches, including the dorsal trunk (Figure 5C). To test whether wild-type *pnt* function was required for specifying extra TCs in *h^674^* mutant trachea, we reduced the gene dosage of *pnt* in *h^674^* homozygous embryos and analyzed TC specification. One copy of *pnt^Δ88^* in *h^674^* homozygous embryos prevented the specification of extra TCs by *h^674^* such that no DBs contained two TCs compared to *h^674^* mutants alone (Figure 5E). These data demonstrate that specification of extra TCs in *h^674^* mutant embryos is dependent on normal *pnt* function. To test whether *pnt* normally acts downstream of *hairy* in restriction of the TC fate, we analyzed embryos mutant for both *hairy* and *pnt*. In *h^674^pnt^Δ88^* double homozygous embryos, no TCs were specified, like in *pnt^Δ88^*, demonstrating that *pnt* likely acts downstream of *hairy* (Figure 5D). Since *pnt* function correlates with specification of the TC fate, we next performed *in situ* hybridization for *pnt* RNA to test whether *pnt* RNA expression was altered in *h^674^* mutant trachea. In *h^674^* heterozygous embryos, 80% of DBs had a single *pnt*-expressing TC and 20% had two *pnt*-expressing TCs (Figure 5F and G). In contrast, in *h^674^* homozygous embryos, only approximately 30% of DBs had a single *pnt*-expressing TC and the remainder of DBs had between two and six *pnt*-expressing TCs per branch (Figure 5F and H). Similar to *h^674^* homozygous embryos, DBs of embryos expressing heat shock induced-*h^ACT^* contained extra *pnt*-expressing DB cells (Figure 5F).

**Figure 6:**
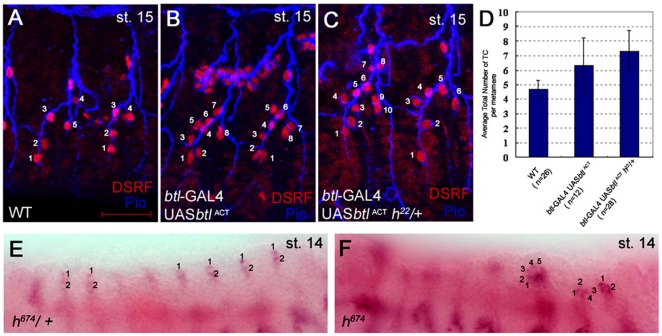
Terminal cell specification by activated breathless is enhanced by loss of hairy. Lateral trunks of wild-type embryos (A) contain approximately five terminal cells (TCs) whereas lateral trunks of trachea expressing activated breathless (*btl^ACT^*) with *btl*-GAL4 (B) and *btl^ACT^* expressing trachea of *h^22^* heterozygous embryos (C) contain more TCs in the lateral trunk branches. Graph shows Hairy-dependent increase in TC specification by activated Btl in metameres 2–9; n represents number of embryos scored (D). In *h^674^* heterozygous embryos (E), each dorsal branch has two cells expressing *btl* RNA whereas in homozygous siblings (F), each dorsal branch has two or more cells expressing *btl* RNA. Embryos in A–C were stained for DSRF (red), Piopio (blue) to label the tracheal lumen and β-gal (not shown). Panels A–C are lateral views of projected confocal images. Embryos in E and F were hybridized *in situ* for *btl* RNA (purple) and *trachealess* RNA (pink). Scale bar in A represents 20 µm.

Tracheal-specific overexpression of an activated form of Btl (Btl^ACT^) or, ubiquitous overexpression of its ligand, Branchless (Bnl) results in an increased number of TCs (Figure 6B) [5]. Therefore, we tested whether the specification of extra TCs observed in *hairy* mutant trachea could be due at least in part to hyper-activation of the Bnl/Btl signaling pathway. Concomitant reduction of *hairy* gene dosage in wild-type trachea already expressing Btl^ACT^ resulted in the specification of more TCs than with Btl^ACT^ alone (Figure 6B, C and D). Moreover, in *h^674^* mutant DBs, *btl* RNA expression was not restricted to the distal most two cells as normally occurred in wild-type DBs and instead was expressed in more proximal cells of the DB (Figure 6E and F). Thus, by reducing *hairy* function, domain of *btl* RNA expression was expanded to include not only the tip cells but also the stalk cells. Altogether, these data suggest that *hairy* normally limits *btl* expression domain and terminal cell specification.

**Figure 7:**
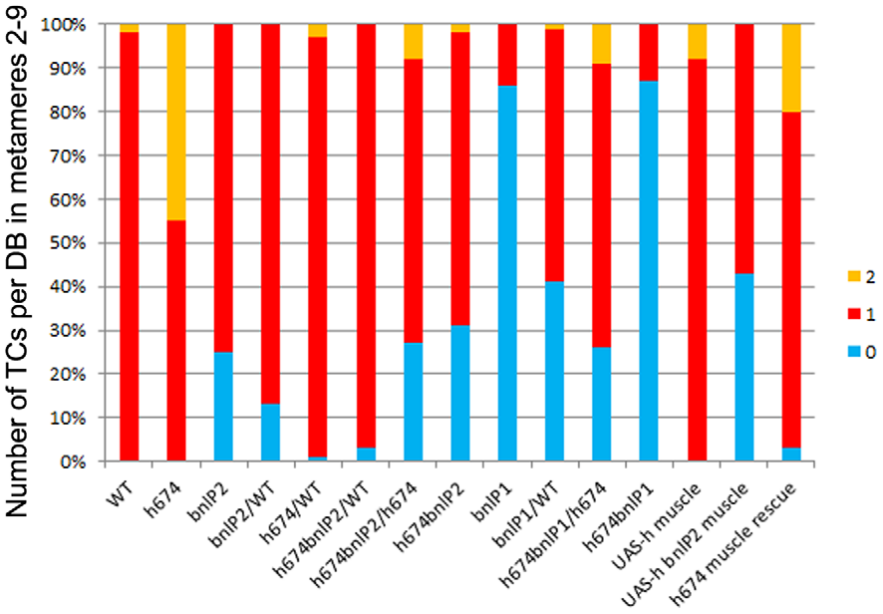
*hairy* functions in the muscle and genetically interacts with *bnl* to refine the terminal cell fate. Graph shows number of DSRF-labeled terminal cells per dorsal branch in metameres two to nine of wild-type, *hairy* and *bnl* mutant embryos. Between 36 and 106 terminal branches were scored for the various genotypes.

Pnt expression is known to be regulated by the *bnl*/*btl* signaling pathway in a positive feed-back loop [12]. Thus, we tested whether *hairy* also genetically interacts with *bnl*. Bnl is known to be haploinsufficient for tracheal branch migration [6]; however, whether it is also haploinsufficient for TC specification has previously not been tested. In embryos with one copy of either *bnl^P1^* or *bnl^P2^*, about 40% and 12% of DBs had no TCs, respectively, compared to embryos homozygous for *bnl^P1^* where 85% had no TCs and embryos homozygous for *bnl^P2^* where 25% of DBs had no TCs (Figure 7). Thus, *bnl* is haploinsufficient for TC specification. In *bnl^P2^* heterozygous embryos that were also heterozygous for *h^674^* (*h^674^bnl^P2^*/WT), the percentage of DBs with no TCs was reduced to 3% from 13% in *bnl^P2^* heterozygotes alone suggesting that one copy of *h^674^* is sufficient to partially alleviate the *bnl^P2^* haploinsufficient phenotype. Reduction of *bnl* gene dosage in *h^674^* homozygous embryos reduced the number of DBs with two TCs from 45% in *h^674^* homozygotes alone to 7% in *h^674^* homozygotes with one copy of *bnl^P2^* and 9% in *h^674^* homozygotes with one copy of *bnl^P1^* (Figure 7). Furthermore, in embryos double homozygous for *h^674^* and *bnl^P1^*, 85% had no TCs, a phenotype more like *bnl* than *hairy* mutants. These data demonstrate that *hairy* and *bnl* genetically interact to refine TC specification and that *bnl* promotes TC specification whereas *hairy* normally acts to restrict it.

**Figure 8:**
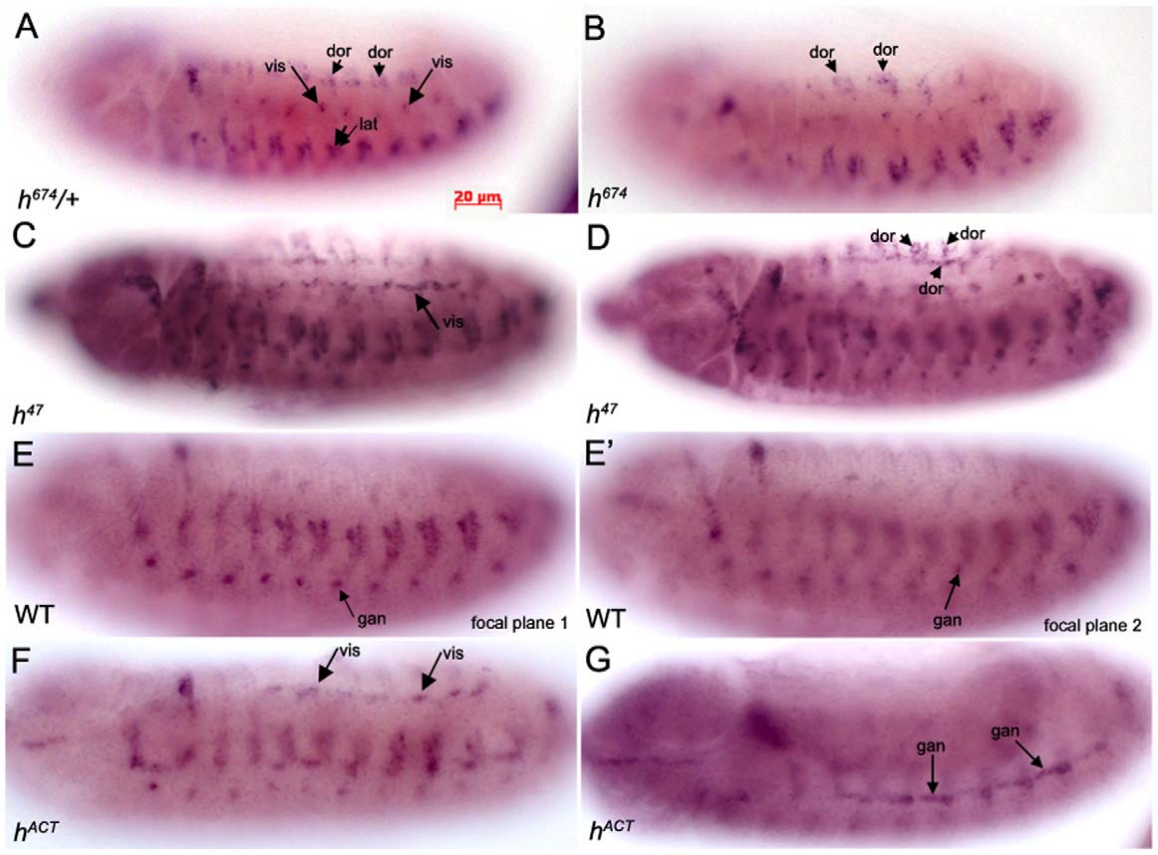
Ectopic expression of *bnl* in *hairy* mutant embryos. In *h^674^* heterozygous embryos (A), *bnl* RNA is expressed in clusters close to the tracheal visceral branch (vis) (A, large arrows), in dorsal clusters (dor) (A, small arrowheads) and in lateral clusters (lat) (A, large arrowhead). In *h^674^* homozygous embryos (B), the dorsal clusters of *bnl* RNA are expanded (B, small arrowheads). In *h^47^* homozygous embryos (C and D), clusters of *bnl* RNA close to the tracheal visceral branch (vis) (C, large arrow) and dorsal clusters (dor) (D, small arrowheads) are expanded. In wild-type embryos (E and E'), a cluster of *bnl* expression is found close to the tracheal ganglionic branch (gan) (E and E', small arrows). In *h^ACT^* embryos (F and G), *bnl* RNA close to the tracheal visceral branch (vis) (F, large arrows) and close to the ganglionic branch (gan) (G, small arrows) are expanded. All embryos shown are at stage 13 and were processed for ISH for *bnl* RNA and *lac-Z* to distinguish *h^674^* heterozygous from homozygous embryos. Embryos in panels A and B are lateral views whereas embryos in panels C–G are ventral-lateral views. Panel E' is a more internal focal plane than that shown in panel E.

Since TC specification occurs after Hairy protein expression disappears in the tracheal cells, we sought to determine whether Hairy could regulate tracheal TC specification in a non cell-autonomous manner. Late expression of *btl* at the tips of growing primary branches is known to be dependent on *bnl* expression in surrounding non-tracheal cells [12]. Since Hairy remains expressed in the somatic and visceral muscle at the stage when TCs are specified (Figure 1), we tested the hypothesis that Hairy may normally regulate TC specification through restriction of *bnl* expression in non-tracheal cells. In wild-type and *h^674^* heterozygous embryos (Figure 8A and E), *bnl* in non-tracheal cells at mid-embryogenesis was expressed in clusters close to the migrating tracheal dorsal branch, visceral branch, lateral trunk and ganglionic branches, as previously reported [6], [12]. We observed expanded domains of *bnl* expression in select groups of non-tracheal cells that were in close proximity to migrating tracheal cells of *h^674^*, *h^47^*and *h^ACT^* embryos (Figure 8). In *h^674^* and *h^47^*mutant embryos, we consistently observed expanded *bnl* RNA expression in the dorsal and visceral clusters (Figure 8B, C and D), whereas in *h^ACT^* embryos, we observed expanded *bnl* RNA expression in the visceral and ganglionic clusters (Figure 8F and G). The proximity of these *bnl* expressing non-tracheal cells to the tracheal branch tips of *h^674^*, *h^47^*and *h^ACT^* mutant embryos likely accounts for the extra TCs observed.

**Figure 9:**
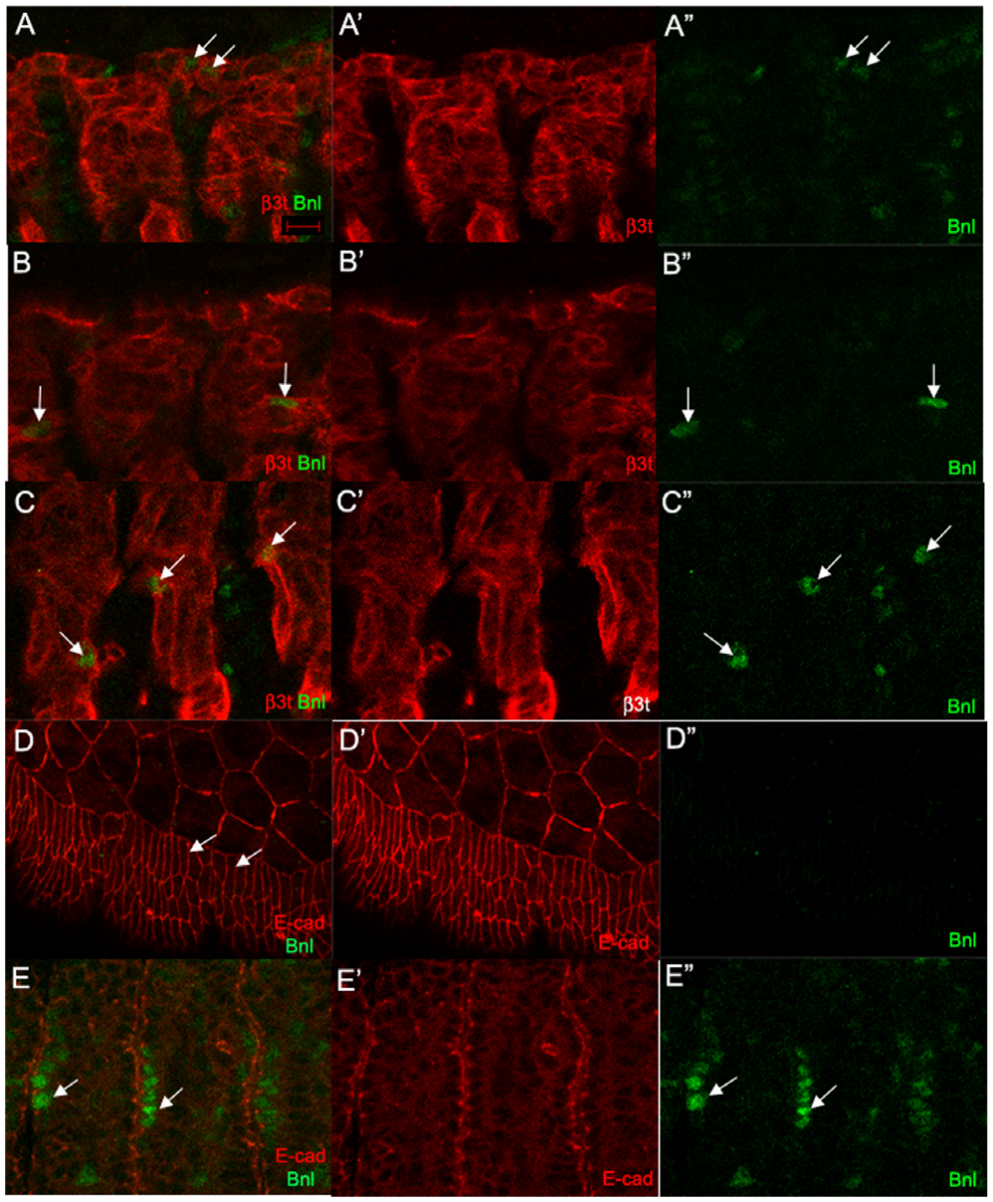
Bnl is expressed in the somatic muscle and ectoderm. In *bnl-lacZ* expressing embryos, (A–E), Bnl (A–E, green) is expressed in the dorsal-most row of muscle cells (A, arrows) and in single cells in the dorsal (B, arrows) and lateral muscles (C, arrows) labeled with β3t (A–C, red). Bnl (D, green) is not expressed in the dorsal epidermis labeled with E-cad (D, arrows, red). Bnl (E, arrows, green) is expressed in the epidermal cells of the segmental grooves labeled with E-cad (E, red). All embryos shown are at stage 14. Embryos in A–C were stained for β3t (red) and β-gal (green) whereas embryos in D and E were stained for E-cad (red) and β-gal. Scale bar in A represents 5 µm.

Using the *bnl^P2^ bnl*-*lacZ* enhancer trap, Bnl was previously reported to be expressed in the visceral muscle and ectodermal clusters [12]; however, the ectodermal expression of Bnl was not confirmed with an ectoderm-specific marker. To better understand the dynamic pattern of Bnl expression during mid-embryogenesis when the TC fate is refined, we analyzed β-galactosidase expression conferred by the *bnl*-*lacZ* enhancer trap together with an ectoderm-specific marker, E-cadherin (E-cad) and a muscle-specific marker, β3 tubulin (β3t). Bnl expression was detected in the most dorsal row of muscle cells as well as in single cells within the somatic muscle which corresponded to the visceral and ganglionic groups of *bnl* RNA expressing cells (Figure 9A-C). We did not detect Bnl in the dorsal epidermis; however, Bnl was robustly expressed in epidermal cells that lined the segmental grooves (Figure 9D and E). Thus, the cells expressing ectopic *bnl* RNA observed in *hairy* mutant embryos corresponded to areas of normal *bnl* expression in the lateral body wall muscle, the same tissue that *hairy* is expressed in.

As previously reported [18], [19] and shown here in Figure 1, Hairy is expressed in the somatic and visceral muscle during mid-embryogenesis. To test whether Hairy refines the TC fate non cell-autonomously, we tested whether *hairy* function was required in the muscle. Expression of wild-type *hairy* in the somatic and visceral muscle did not affect TC specification (Figure 7). Expression of wild-type *hairy* in the somatic and visceral muscle of *h^674^* homozygous embryos reduced the percentage of dorsal branches with two TCs from 45% to 20% and increased the percentage of DBs with one TC from 55% to 80% (Figure 7). Moreover, expression of wild-type *hairy* specifically in the muscle of *bnl^P2^* heterozygous embryos enhanced the haploinsufficient phenotype of *bnl^P2^* by forming more DBs with no TCs compared to *bnl^P2^* heterozygotes alone. These data demonstrate that *hairy* functions at least in part in the muscle to refine the number of TCs specified in the trachea.

**Figure 10:**
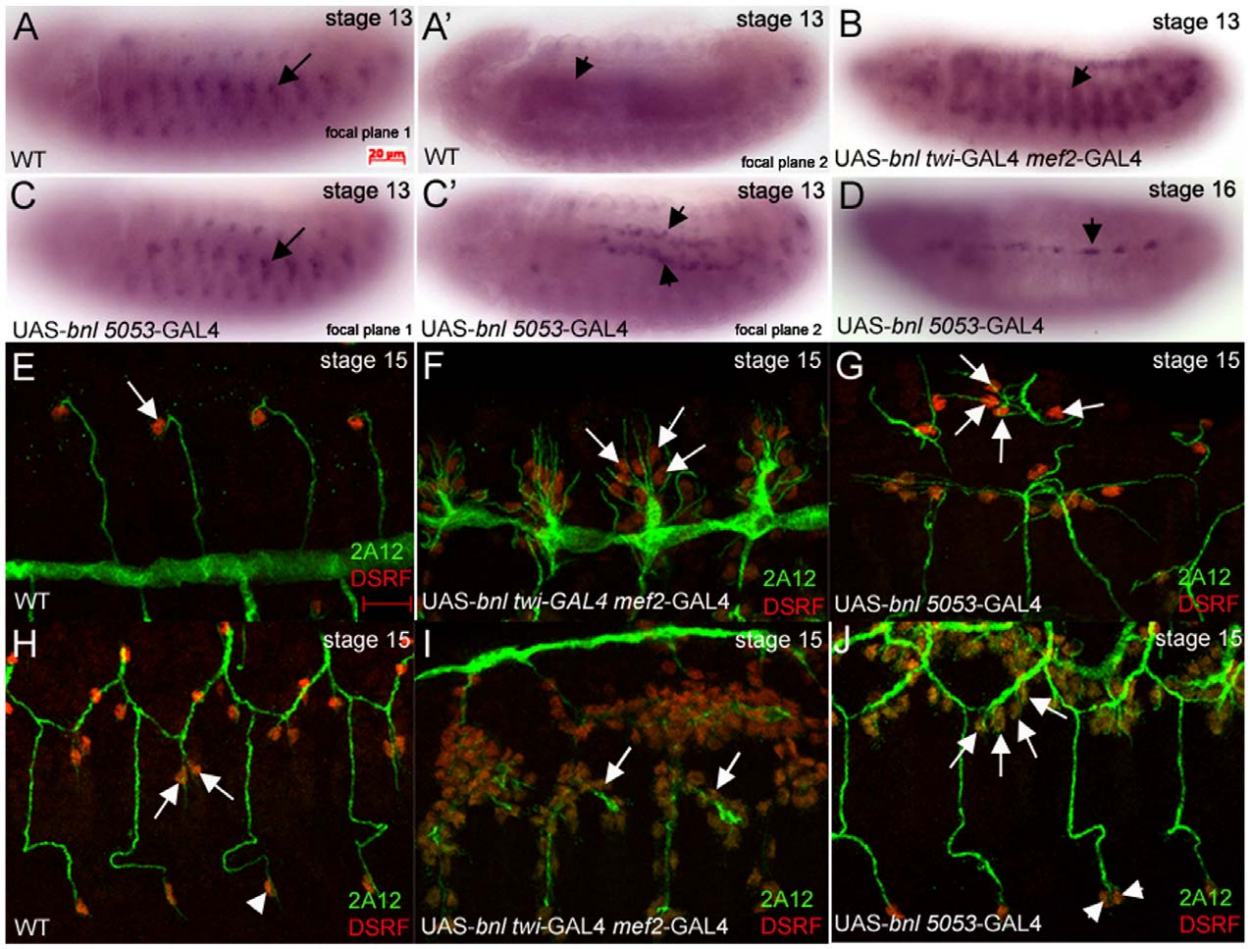
expression of *bnl* in the mesoderm phenocopies *hairy* mutant phenotype by specifying extra terminal cells. In stage 13 wild-type embryos (A and A'), *bnl* RNA is expressed in clusters surrounding the migrating trachea (A, arrow) and to a lesser extent in the circular visceral mesoderm (A', arrowhead). In stage 13 embryos where *bnl* is overexpressed with *twi*-GAL4 *mef2*-GAL4 (B), *bnl* RNA is expressed in the somatic mesoderm (B, arrowhead). In stage 13 embryos where *bnl* is expressed with *5053*-GAL4 (C and C'), *bnl* RNA is expressed in clusters close to the tracheal cells (C, arrow) and in longitudinal muscle founder cells of the visceral mesoderm (C' arrowheads). In stage 16 embryos expressing *bnl* with *5053*-GAL4 (D), *bnl* RNA is expressed in the ventral oblique lateral body wall muscles (D, arrowhead). In wild-type embryos (E), a single terminal cell (TC) is specified at the tip of each dorsal branch (E, arrow). In embryos overexpressing *bnl* in the entire mesoderm with *twi*-GAL4 *mef2*-GAL4 (F) or in a subset of the mesoderm with *5053*-GAL4 (G), extra TCs are specified in the dorsal branches (F and G, arrows). In wild-type embryos (H), a distinct number of TCs are specified in the lateral trunks (H, arrows) and the ganglionic branch (H, arrowhead) whereas in embryos expressing *bnl* in the mesoderm with either *twi*-GAL4 *mef2*-GAL4 (I) or *5053*-GAL4 (J) extra TCs in the lateral trunks (I and J, arrows) and ganglionic branches (J, arrowheads) are specified. Embryos in A–D were hybridized *in situ* with *bnl* RNA. Embryos in E–J were stained for DSRF (red) and 2A12 (green). All panels shown are lateral views except panel G which is a dorsal-lateral view. Scale bar in E represents 10 µm.

Since our data demonstrated a role for *hairy* in the muscle, we next tested whether overexpression of *bnl* in the muscle can phenocopy the *h^674^*, *h^47^*and *h^ACT^* mutant phenotype of extra TC specification. We expressed wild-type *bnl* in the somatic and visceral muscle with *twi-*GAL4 *mef2*-GAL4, or in a subset of the visceral muscle and the ventral longitudinal muscle 1 with *5053*-GAL4 (Figure 10A–D). Overexpression of *bnl* in the muscle led to formation of DBs that consisted almost entirely of TCs (Figure 10F); extra TCs were also specified in the transverse connective and the lateral trunk (Figure 10I). Overexpression of *bnl* with *5053*-GAL4 phenocopied the *hairy* mutant phenotype more closely where a few extra TCs were specified in the dorsal, lateral trunk and ganglionic branches (Figure 10G and J). These data demonstrate that overexpression of *bnl* in the somatic and visceral muscle is sufficient to phenocopy the *hairy* mutant phenotype.

### Hairy controls terminal branch lumen length in a *bnl*-dependent manner

**Figure 11:**
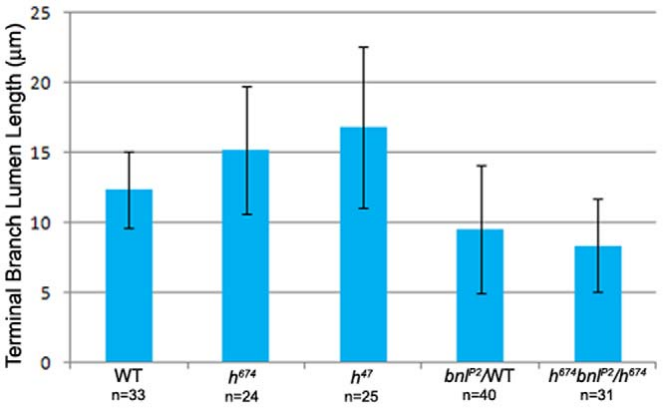
Hairy and Bnl control terminal cell lumen length. Graph showing lumen length of terminal branches in wild-type embryos, *h^674^* and *h^47^* homozygous embryos, *bnl^P2^* heterozygous embryos and *h^674^*homozygous embryos with one copy of *bnl^P2^*. n =  number of terminal branches scored. Values are means ± S.D. *P*<0.001.

In addition to refinement of the TC fate, Hairy also restricts terminal branch lumen length. Quantification of terminal branch (TB) lumen length (see Materials and Methods) showed longer lumens in *h^674^*and *h^47^* homozygous embryos compared to wild-type embryos (Figure 11). TB lumen length of *h^674^*and *h^47^* homozygous embryos measured 15±4.5 µm and 17±6 µm, respectively, compared to 12±3 µm in wild-type embryos. Extension of TB lumen length is dependent on *bnl* expression since loss of one copy of *bnl^P2^* in otherwise wild-type embryos or in *h^674^* homozygous embryos reduced TB lumen length to less than that of wild-type embryos (Figure 11).

## Discussion

Hairy plays a well characterized role in patterning of the early *Drosophila* embryo; however, our knowledge of *hairy* function in epithelial morphogenesis is limited to our previous study on *hairy*'s role in the regulation of apical membrane growth during embryonic salivary gland development. In this study, we demonstrate a novel function for *hairy* in refinement of the terminal cell fate to a single cell at the tip of the dorsal branch through restriction of *bnl* expression domains in muscle cells surrounding tracheal cells. Due to the strong requirement for *hairy* in early embryonic patterning, it was necessary to distinguish *hairy* function in tracheal development from its earlier patterning role. Thus, we focused our analysis of *hairy* function in the trachea to mutations that did not perturb segmentation of the embryo. We showed that as previously reported, *h^47^* mutant embryos had no patterning defect, and yet, extra TCs were specified. *h^ACT^*, which not only lacks the WRPW motif but also contains the transcriptional activation domain of VP16, was previously shown to induce ectopic expression of target genes when expressed in the late blastoderm stage, the time when endogenous Hairy is expressed and is active [31]. We showed that expression of *h^ACT^* in mid-embryogenesis, prior to specification of the terminal cell fate led to ectopic expression of *bnl* in muscle cells and specification of extra TCs. Since *h^ACT^* was induced after patterning of the early embryo was complete, there were no segmentation defects in *h^ACT^*-expressing embryos and yet, extra TCs were specified. Similar to *h^ACT^*, in *h^674^* mutant embryos with no segmentation defect, *bnl* expression domain was expanded and extra TCs were specified. Since, *h^674^*, like *h^ACT^*, lacks the C-terminal co-repressor binding WRPW tetrapeptide [26], [31]it is possible that the Hairy mutant protein of *h^674^* embryos also acts as an activator and induces expression of downstream target genes. Thus, the *h^47^*, *h^674^*and *h^ACT^* mutant embryos which are segmented properly and yet show specification of extra TCs in the tracheal dorsal branches provide evidence that *hairy*'s role in early patterning and in tracheal development are indeed distinct.

In addition to a role for *hairy* in refinement of the terminal cell fate through regulation of *bnl* expression in muscle cells, we also provide evidence that *hairy* and *bnl* act antagonistically to regulate terminal branch lumen length. Our studies provide the first evidence for a role for *bnl* in tracheal lumen size control. Although *hairy* mutant tracheal cells invaginated completely, they did so in an uncoordinated manner compared to wild-type. Thus, *hairy* function is required at multiple stages of tracheal development.

Our data support a model where Hairy in the somatic muscle, normally refines the spatial expression of *bnl* in the muscle cells that are in close proximity to the migrating tracheal branches, such that only a single cell at the tip of each dorsal branch becomes specified as the terminal cell. Upon loss of Hairy's repressive activity, *bnl* expression expands in the muscle cells and abnormally activates the *bnl*/*btl* signaling pathway, such that extra TCs become specified. Our data do not suggest whether *bnl* is a direct or indirect transcriptional target of Hairy; future studies will distinguish between these two possibilities. It is also possible that Hairy regulates *bnl*/*btl* signaling and TC specification via mechanisms other than control of *bnl* expression. For example, it was recently shown that in the developing tracheal air sac of *Drosophila* larvae, matrix metalloprotease Mmp2 spatially restricts FGF signaling [32]. Thus, *hairy* may modulate the extent of *bnl*/*btl* signaling in tracheal cells in a post-translational manner as well.

## Materials and Methods

### *Drosophila* Strains and Genetics

Canton-S (CS) flies were used as wild-type controls. The following fly lines were obtained from the Bloomington Stock Center and are described in FlyBase (http://flybase.bio.indiana.edu/): *h^22^, h^1^*, *h^1J3^*, *pnt^Δ88^*, *twi*-GAL4, *mef2*-GAL4 and *505*3-GAL4. *h^674^* was generated by standard EMS mutagenesis [26]. *h^ACT^, h^47^*, *h^m2^*and UAS-*hairy* were generous gifts of M. Wainwright and D. Ish-Horowicz. For the UAS-GAL4 expression system [33], *btl*-GAL4 was used to drive tracheal specific expression of wild-type *hairy* (UAS-*hairy*) or activated *btl* (UAS-*btl^ACT^*) and *twi*-GAL4 *mef2*-GAL4 and *5053*-GAL4 were used to drive expression of wild-type *bnl* (UAS-*bnl*) or wild-type *hairy*.

### Antibody Staining of Embryos

Embryo fixation and antibody staining were performed as previously described [34]. The following antisera were used at the indicated dilutions: mouse 2A12 antiserum (Developmental Studies Hybridoma Bank, DSHB; Iowa City, IA) at 1∶5 for DAB staining and 1∶2 for fluorescence; mouse Crumbs antiserum (DSHB) at 1∶10; rabbit Trachealess antiserum (a gift from M. Llimargas) at 1∶30; mouse DSRF antiserum (Active Motif, Carlsbad, CA) at 1∶100; mouse β-galactosidase (β-gal) antiserum (Promega, Madison, WI) at 1∶10,000 for DAB staining and 1∶500 for fluorescence; rat Hairy antiserum (a gift from S. Small) at 1∶1; rabbit β3 tubulin (β3t) antiserum (a gift from R. Renkawitz-Pohl) at 1∶10,000 and rat E-cadherin (E-cad) antiserum (DSHB) at 1∶20. Appropriate biotinylated- (Jackson Immunoresearch Laboratories, Westgrove, PA), AlexaFluor488-, 647- or Rhodamine- (Molecular Probes, Eugene, OR) conjugated secondary antibodies were used at a dilution of 1∶500. Whole-mount DAB stained embryos were mounted in methyl salicylate (Sigma, St. Louis, MO) and embryos were visualized on a Zeiss Axioplan 2 microscope with Axiovision Rel 4.2 software (Carl Zeiss, Thornwood, NY). Whole-mount immunofluorescent stained embryos were mounted in Aqua Polymount (Polysciences, Inc., Warrington, PA) and thick (1 µm) fluorescence images were acquired on a Zeiss Axioplan microscope (Carl Zeiss) equipped with LSM 510 for laser scanning confocal microscopy at the Rockefeller University Bio-imaging Resources Center (New York, NY) and the Weill Medical College imaging research core facility.

### RNA In Situ Hybridization

*In situ* hybridization (ISH) with antisense digoxigenin-labeled RNA probes for *hairy* (*h*), *spalt* (*sal*), *breathless* (*btl*), *branchless* (*bnl*), *pointed* (*pnt*) and *β-galactosidase* (*β-gal*) were performed as previously described [35]. *h*, *sal*, *btl*, *bnl*, *pnt* and *β-gal* cDNAs were used as templates for generating antisense digoxygenin-labeled RNA probes as previously described [26]. Embryos were mounted in 70% glycerol before visualization as described above for whole mount antibody staining.

### Heat shock induction of Hairy^ACT^

Embryos were collected for 8 hours on apple juice agar plates and then subjected to heat shock at 37°C for 10 minutes in a dry incubator. The embryos were then aged for a further 8 hours at room temperature (25°C or 16 hours at 18°C). Stage 15 embryos were prepared for immunocytochemistry or whole mount *in situ* hybridization as described above.

### Quantification of terminal branch lumen length

Terminal branch lumen length was measured as the distance between center of terminal cell nucleus and tip of 2A12-labeled lumen and was measured with LSM 510 software. N represents the total number of TCs scored. Statistical analysis was completed using Microsoft Excel (Microsoft, Redmond, WA). P-values were calculated using Student's two-tailed, unpaired *t*- tests.
